# The haematoma and its role in bone healing

**DOI:** 10.1186/s40634-017-0079-3

**Published:** 2017-02-07

**Authors:** H. Schell, G. N. Duda, A. Peters, S. Tsitsilonis, K. A. Johnson, K. Schmidt-Bleek

**Affiliations:** 1 Julius Wolff Institut and Center for Musculoskeletal Surgery Charité - Universitätsmedizin Berlin, Berlin, Germany; 2 Berlin-Brandenburg Center for Regenerative Therapies, Charité – Universitätsmedizin Berlin, Berlin, Germany; 3 Center for Musculoskeletal Surgery, Charité – Universitätsmedizin Berlin, Berlin, Germany; 4 Faculty of Veterinary Science, University of Sydney, Sydney, Australia

**Keywords:** Haematoma, Bone healing, Regeneration, Fracture treatment

## Abstract

Fracture treatment is an old endeavour intended to promote bone healing and to also enable early loading and regain of function in the injured limb. However, in today’s clinical routine the healing potential of the initial fracture haematoma is still not fully recognized. The Arbeitsgemeinschaft für Osteosynthesefragen (AO) formed in Switzerland in 1956 formulated four AO principles of fracture treatment which are still valid today. Fracture treatment strategies have continued to evolve further, as for example the relatively new concept of minimally invasive plate osteosynthesis (MIPO). This MIPO treatment strategy harbours the benefit of an undisturbed original fracture haematoma that supports the healing process. The extent of the supportive effect of this haematoma for the bone healing process has not been considered in clinical practice so far. The rising importance of osteoimmunological aspects in bone healing supports the essential role of the initial haematoma as a source for inflammatory cells that release the cytokine pattern that directs cell recruitment towards the injured tissue. In reviewing the potential benefits of the fracture haematoma, the early development of angiogenic and osteogenic potentials within the haematoma are striking. Removing the haematoma during surgery could negatively influence the fracture healing process. In an ovine open tibial fracture model the haematoma was removed 4 or 7 days after injury and the bone that formed during the first two weeks of healing was significantly reduced in comparison with an undisturbed control. These findings indicate that whenever possible the original haematoma formed upon injury should be conserved during clinical fracture treatment to benefit from the inherent healing potential.

## Review

### Fracture treatment: a retrospective reflection

Fracture healing is a well-orchestrated process involving the interplay of multiple cell types, various cytokines, chemokines, and growth factors which can result in reconstituted bone without any scar tissue. However, despite the great developments in fracture management that have been made in the last century, the healing sequence is vulnerable, as even today the outcome in up to 10% of fractures is unsatisfying for the patient and the clinician (Dimitriou et al. 2005; Haas 2000). The standard treatment of fractures by coaptation and limb traction with bed rest, which resulted in many cases in muscular atrophy, joint contractures and poor functional outcome, began to be seriously questioned with the introduction of intramedullary nailing of femoral fractures by Kűntscher in Germany in the 1940s, and then later the formation of the Arbeitsgemeinschaft fűr Osteosynthesefragen (AO) in Switzerland in 1956 (Heim 2001). The four AO principles of fracture treatment that were directed towards early return to full function were the following: 1. Accurate anatomical reduction especially of intra-articular fractures, 2. Atraumatic operative technique preserving the vitality of bone and soft tissues, 3. Rigid internal fixation, and 4. Avoidance of soft tissue damage and so-called “fracture disease” (Müller et al. 1979). An integral part of the AO mission has been the teaching of internal fixation techniques often using plastic bone models. Understandably, this method of teaching focused on the first and third AO principles, and the preservation of soft tissues espoused by the second principle was often overlooked in the plastic bone exercise and later in the surgical operating room. Moreover during this early period, the quest to achieve complete anatomical fracture reduction, interfragmentary compression and callus-free primary bone union became the principle aspiration for the fracture surgeon. During such surgical procedures, the fracture haematoma was simply flushed away to allow a better view of the fracture fragments, thus allowing absolute anatomical reduction with interfragmentary compression and therefore primary fracture healing. Indeed, in the 1980s, the importance of the fracture haematoma was controversially discussed. One view was that the fracture haematoma was a hindrance to union, while others considered that the fibrin network of the blood clot acted as a scaffold for fibrocellular invasion, and hence was beneficial to fracture repair (Sevitt 1981).

The introduction of the concept of minimally invasive plate osteosynthesis (MIPO) around the turn of this century was founded on the emerging body of evidence that both the bone blood supply and the fracture haematoma should be preserved to optimize the fracture healing process, especially in cases of extra-articular diaphyseal fractures (Tong and Bavonratanavech 2007; Wagner and Frigg 2006). This approach to management of closed diaphyseal fracture in clinical practice has been greatly facilitated by the widespread introduction of locking bridging plates, as well as new locked nailing technology that respects periosteal blood perfusion. Relative fracture stability can be achieved with a bridging locked plate fixation, leading to secondary bone union with callus formation. One advantage of locked plates is that they do not need to be accurately contoured to the surface of the underlying bone because the construct stability is largely due the angular stability of the locked screws, and it does not rely on friction between the undersurface of the plate and the bone as is the case with conventional bone plates such as the limited contact dynamic compression plates (Babst et al. 2012). Consequently it has been proposed that locked plates produce less disruption of periosteal blood flow in the underlying cortical bone, although this has not been definitely demonstrated experimentally. However, the effects of locked plates and conventional bone plates on the fracture haematoma per se are likely to be similar, and depend more upon the surgical techniques used for fracture reduction and implant placement (Tong and Bavonratanavech 2007). Both locking plates and the limited contact dynamic compression plates can be applied with compression plates function (absolute stability) or bridging plate function (relative stability), depending on the degree of fracture comminution.

In the context of the importance of the haematoma in fracture repair the reaming procedure should be considered as it is associated with increased bleeding. Reaming of the medullary canal of the femur for autologous bone graft collection using the reamer-irrigator-aspirator creates a cavity that fills with haematoma due to the extensive disruption of the medullary arterial vasculature. Magnetic resonance imaging studies of patients at 3 months following this procedure have demonstrated intense vascularization of the endosteal surface of the cortex, with new blood vessels progressing circumferentially from the periphery of the haematoma, centrally into the canal (Rankine et al. 2015). This was subsequently followed at 14 months or more by reformation of normal intramedullary bone. In the case of diaphyseal fractures of long bones, intramedullary reaming prior to intramedullary nailing is primarily performed for mechanical reasons; insertion of a larger diameter nail increases stability of the fracture repair construct. However, intramedullary nailing following reaming was shown to impair vascular perfusion acutely in approximately 70% of the tibial cortex of experimental dogs (Klein et al. 1990). Furthermore, the immediate insertion of an intramedullary nail following reaming apparently tamponades the ruptured intramedullary vessels, preventing the formation of a large intramedullary haematoma as seen with the reamer-irrigator-aspirator procedure (Rankine et al. 2015). As a consequence, restoration of intramedullary ossification following fracture union and nail removal is considered to be less likely in the longer term (Rankine et al. 2015).

The purpose of this review is to examine the current knowledge about the importance of the fracture haematoma in bone healing. This review will be completed by original research data on the effect of fracture haematoma removal during open fracture reduction. These data support the relative importance of the fracture haematoma in secondary bone union.

## When should a haematoma be kept intact in current clinical treatment concepts?

**Fig. 1:**
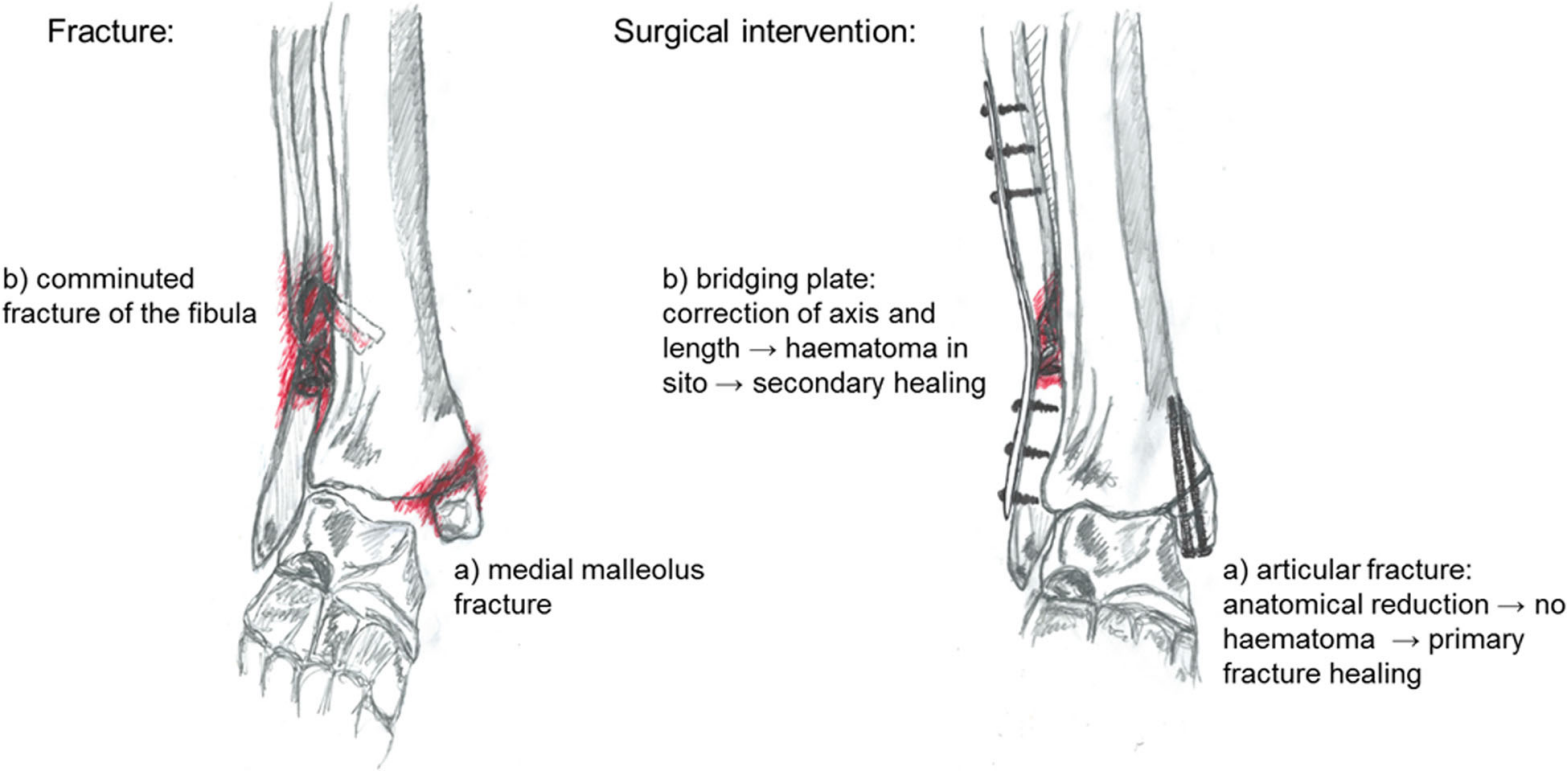
In a comminuted fracture of the fibula and a medial malleolus avulsion fracture the standard of care would include an internal plate fixation of the fibula fracture without the elimination of the fracture haematoma. The bone fragments would remain embedded in the ensuing haematoma while the plate fixation would ensure maintenance of the correct axis and length of the healing fibula. On the other hand leaving the haematoma in the medial malleolar fracture depicted here is not feasible. Here the fracture ends have to be repositioned through an anatomical reduction and this includes the removal of the haematoma. This is of special importance as a joint is involved in the fracture and the correct realignment of the bones of the joint has to be ensured

In the case of reconstructable intra-articular fractures, the AO-principles of open reduction with anatomical realignment and rigid stability will include the complete removal of the haematoma (Fig. 1a). This treatment aims at primary bone healing with little or no callus formation to preserve the synovial joint mobility. If, however, the reduction is not anatomical or the fixation allows relative interfragmentary movements, any consecutive bleeding will lead to a second, less competent haematoma with inferior biological quality. In such cases, fracture healing is both biologically and mechanically challenged and the risk of delayed or non-union drastically increases.

In extra-articular diaphyseal fractures the haematoma, as well as all other soft tissue structures, can be kept in place and must not be removed. The restoration of bone length, and axial and torsional alignment in these cases is more important than anatomical reduction of the fracture (Fig. 1b). In these cases the principle of minimally invasive treatment (e.g. percutaneous bridging plate) would conserve the haematoma and thus an important contributor to the healing process.

## Phases of bone healing: early critical role of fracture haematoma

**Fig. 2:**
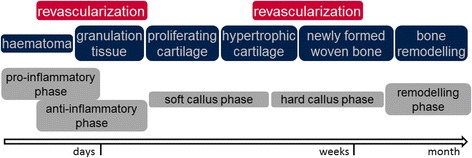
The different tissues involved in bone regeneration are shown above with the two important revascularization steps during tissue development. In the lower line the 5 consecutive phases of bone healing are depicted

Each healing process starts with haematoma formation (Fig. 2) in the inflammatory phase, simply resulting from blood vessel disruption upon injury and fracturing of the bone (Opal 2000). Inherently, the initial phase of the healing process is considered to be one of the critical determinants of the healing outcome (Kolar et al. 2010). With the onset of inflammation, regeneration begins by migration of mesenchymal stem cells, endothelial cells, and immune cells towards the fractured bone region (Kolar et al. 2010; Oe et al. 2007; Schmidt-Bleek et al. 2012a). An important feature of bone healing seems to be the tight regulation of the inflammatory phase. The upregulation of anti-inflammatory signalling coincides with the upregulation of angiogenic factors in the bone haematoma (Schmidt-Bleek et al. 2012a; Schmidt-Bleek et al. 2015). Within the first four days after trauma, the fracture haematoma develops an osteogenic potential that enables bone formation in ectopic locations when explanted and consequently secondary fracture healing (Mizuno et al. 1990). In human fracture haematoma, an upregulation of the osteogenic factors SPP1 (secreted phosphoprotein 1 / osteopontin) and RunX2 (runt-related transcription factor 2) observed within the first 72 h supports the fact that bone formation is already initiated during the haematoma phase of fracture healing (Kolar et al. 2011; Schmidt-Bleek et al. 2012a). Interestingly the expression of bone morphogenetic proteins (BMP) 2, 4, and 7 are relatively low in the initial healing period when compared with later healing phases (woven bone formation, remodelling) (Lienau et al. 2010). BMP 2 and 7 are the growth factors that have been clinically approved for the treatment of non-unions. There they are administered after debridement and refreshing of the fracture ends, often in combination with autologous bone grafts, during the newly created initial pro-inflammatory phase of bone healing. Hereby these growth factors are being administered in a supraphysiological concentration with a scaffold that enables a burst release during this early healing phase. Amazingly recent studies imply that a lower concentration would be even more effective (reviewed in Schmidt-Bleek et al. 2016).

In addition to its osteogenic potential, fracture haematoma also develops an angiogenic potential (Schmidt-Bleek et al. 2012a; Street et al. 2000) with revascularization of the injured region being a prerequisite for the continuance of the healing process (Schmidt-Bleek et al. 2015). Previous studies determined two time points during bone healing to be decisive for revascularization: An early time point around day 7 (at the end of the inflammatory phase) and a later time point around day 21 (beginning of the woven bone formation) (Fig. 2) (Lienau et al. 2009). During the early phase VEGF (vascular endothelial growth factor) is upregulated (Lienau et al. 2009), which is in concurrence with the high VEGF concentrations in human fracture haematoma (Street et al. 2000). This early upregulation of VEGF is conclusive as hypoxia induced angiogenesis is mediated by VEGF. Amazingly immune cells are potent producers of VEGF (e.g. macrophages, dendritic cells). This interaction of the inflammatory and revascularization step during the early phase of healing has previously been reviewed: Schmidt-Bleek et al. 2015.

The mesenchymal, endothelial and immune cells entering the haematoma become embedded and generate the extracellular matrix that evolves into granulation tissue before the soft callus emerges. Cells and matrix are interdependent; cells organize their matrix, whereas the matrix provides the microenvironment for transmitting signals governing cellular functions, such as proliferation, differentiation, and migration (Hutmacher et al. 2012). The adjacent tissues, in particular the bone marrow, are the source for factors or cells invading the region of the injured bone. Studies have shown that the bone marrow composition is altered in the vicinity of a fracture, both in terms of cell composition and factor pattern (Kolar et al. 2011; Konnecke et al. 2014; Schmidt-Bleek et al. 2012a). These changes are time dependent; the tissue surrounding the fracture haematoma matures during the early bone healing phase, supporting the healing process in a tightly correlated and sequentially determined course. During the first days, the haematoma starts to organize and serves as a fibrin network for the migration and proliferation of osteogenic and chondrogenic progenitor cells (Brighton 1984). Cells and soluble molecules within the haematoma initiate the cascade of events essential for fracture healing (Bolander 1992). The progenitor cells, originating from the periosteum, the bone marrow, and the surrounding tissue (Yellowley 2013) react to the signals sent by the haematoma and migrate into the fracture area, thus initiating the soft callus phase of endochondral bone healing. The transformation into a hard callus depends on remodelling of proliferating cartilage into hypertrophic cartilage, the revascularization of this region, the onset of matrix mineralization and woven bone formation. The stability of the fractured region is increased (increased moment of inertia) to a certain degree through hard callus formation (Bucher et al. 2016). However, the mechanical properties of the bone have to be restored during the last step of successful bone healing, namely the remodeling phase. This remodelling phase can span several months, until form and function of the bone are rebuilt (Bucher et al. 2016).

## Duplicity of cytokines

The haematoma represents the beginning of the healing process. The conditions however are not ideal for cells and thus only specific cells are active in this healing stage (Street et al. 2000). Among these cells are macrophages and T cells and these cells secrete a cytokine pattern which in consequence orchestrates the healing (Gaber et al. 2005; Gaber et al. 2009; Hoff et al. 2013). In consequence the field of osteoimmunology becomes more important in the understanding of bone regeneration, and subsequently, inflammatory cytokines are considered as possible therapeutic targets for bone regeneration enhancement. In a recent study we showed the negative effect of terminally differentiated CD8 positive T cells on bone healing (Reinke et al. 2013). The negative effect of these immune cell subset has been linked to the ratio of TNFα (tumor necrosis factor alpha) and IFNγ (interferon gamma), two pro-inflammatory cytokines. The implementation of inflammatory cytokines, however, proves to be difficult when considered for therapeutical approaches (Mountziaris et al. 2011). This is probably due to the tight regulation of these factors during the normal bone healing sequence. TNFα has been described to play an important role during the early phase of healing, where its expression is upregulated. After this point, TNFα is not necessary for a certain period during the healing cascade and thus down regulated before it is upregulated once again to support bone formation during a later healing phase (Gerstenfeld et al. 2003). An example for the critical balance of inflammatory cytokines during the healing process is the TNFα concentration: the absence of TNFα delays fracture healing, while a prolonged higher TNFα concentration destroys bone (Karnes et al. 2015; Mountziaris et al. 2011). Interleukin 17 (IL-17), which is the lead cytokine of T helper 17 cells, for example, has catabolic effects (by enhancing osteoclasts), as well as anabolic effects (supporting osteoblasts) (Nam et al. 2012; Takayanagi 2007). These opposing effects show that the expression is adapted to resprective healing phases. In contrast, IFNγ has been reported to hinder osteoclastogenesis (Arron and Choi 2000; Takayanagi et al. 2000) as well as osteoblastogenesis (Cornish et al. 2003). Simultaneous stimulation/ inhibition of both, bone degradation and bone formation, is not supportive for regeneration. Each healing phase therefore needs a tightly regulated signalling pattern to balance cytokines present during certain healing stages.

## Towards regeneration or scar formation

The synergistic interaction of bone cells and immune cells, especially during the process of regeneration, is still largely unknown even though some aspects have been investigated (Lorenzo et al. 2011). A balanced immune response appears to be essential for a successful bone healing process (Kolar et al. 2010; Schmidt-Bleek et al. 2012a; Schmidt-Bleek et al. 2012b) - and this balance, so far, is best achieved in the haematoma ensuing upon injury. The unique property of bone to regenerate is highly dependent of these early processes during healing (Harty et al. 2003). The provisional matrix, the fibrin clot, formed upon clotting harbors proteases, growth factors and cytokines governing cellular actions (Schaffer and Nanney 1996). The cells predominately active during this healing stage are neutrophils, leucocytes, and macrophages which initiate the first changes in the early matrix by activating fibroblasts which in consequence release hyoluronate and glycoporteins (fibronectin) into the developing extra cellular matrix (ECM) (Singer and Clark 1999). Upon healing progression a granulation tissue develops further changing the ECM by secretion of proteoglycans and collagens. It is at this early stage of the healing process that the fate of scarring (mainly repairing by creating a replacement tissue) and regeneration (restoring form and function) seems to be determined. This becomes apparent when fetal wound healing (no scarring) is compared with adult wound healing (scarring). A reduced vascularity and macrophage infiltration is observed in the fetal wounds when compared with adult wounds. However, in case of a severe wound with localized necrosis, the macrophage infiltration is enhanced in fetal wound and scarring occurs (Hopkinson-Woolley et al. 1994). This highlights the central role of the early inflammatory reaction in the haematoma in determining the healing outcome as a crossroad towards regeneration or scar formation (Harty et al. 2003).

## The early haematoma determines the fracture healing progress

**Fig. 3:**
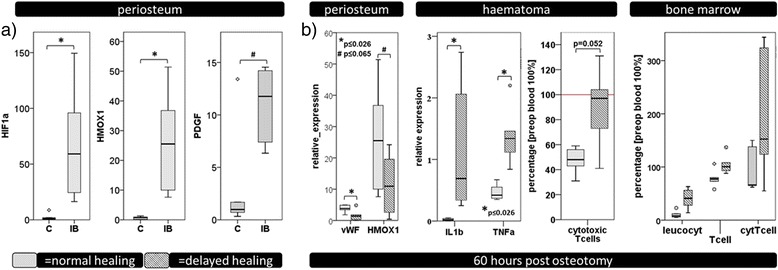
**a** Upon injury the cytokine expression changes in the periosteum directly adjacent to the bone injury: C = uninjured control, IB = injured bone 60 h after injury. HIF1a, HMOX1, and PDGF, three factors highly relevant for revascularization are significantly upregulated in the periosteum upon injury. **b** Tissues involved in bone healing, periosteum, haematoma, and bone marrow were investigated under normal and delayed healing conditions. The cytokine expression and also the cellular composition in all compartments was significantly altered under mechanically induced delayed healing conditions. For detailed information please refer to: (Schmidt-Bleek et al. 2012a; Schmidt-Bleek et al. 2012b). For statistical analyses of data, medians were calculated for each group per time point. Statistical comparisons between the groups were performed using the Mann–Whitney *U*-test (SPSS 22; SPSS, Inc., Chicago, IL)

The first steps of the healing process proceed during the haematoma maturation. A certain cytokine pattern is established that is essential in guiding the cell recruitment towards the injury site but also in coordinating the cellular activity of those cells (Hankenson et al. 2015). An example for this coordination is the change from the macrophages with a pro-inflammatory phenotype (M1 macrophages) that are present in the initial fracture haematoma towards macrophages with a M2 phenotype during the first 3 days of healing (Schlundt et al. 2015; Sinder et al. 2015). This switch could be regulated by a change in cytokines towards interleukin 4 and 13. Previous studies have shown that the injury activates the expression of factors important for the healing process. In the periosteum adjacent to the fractured bone for example hypoxia induced factor 1 alpha, heme oxygenase-1 and platelet derived growth factor are significantly upregulated (Fig. 3a) (unpublished data). The importance of the cytokine pattern can be deduced from the fact that 60 h after bony injury the expression of relevant factors for inflammation and revascularization as well as the cellular composition in all the tissues concerned in bone healing (periosteum, fracture haematoma and bone marrow) are distinctly changed when a normal healing situation is compared with a mechanically induced delayed healing situation (Fig. 3b) (Schmidt-Bleek et al. 2012a; Schmidt-Bleek et al. 2012b).

**Fig. 4:**
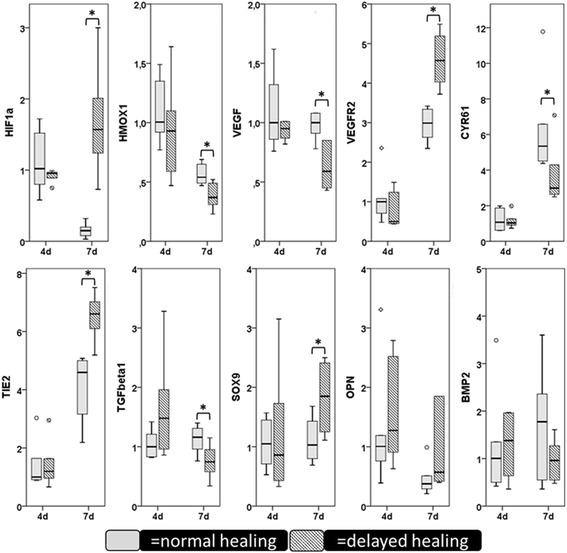
The expression profile of factors involved in inflammation, angiogenesis and osteogenesis change with the progression of healing, e.g. from 4 to 7 days, in the fracture haematoma and is also dependent of the healing progression. The cytokine pattern in a normal, undisturbed healing differs from one in a mechanically induced delayed healing. For statistical analyses of data, medians were calculated for each group per time point. Statistical comparisons between the groups were performed using the Mann–Whitney *U*-test (SPSS 22; SPSS, Inc., Chicago, IL). (For a detailed description of these experiments please consult (Lienau et al. 2009; Lienau et al. 2010; Schmidt-Bleek et al. 2012a; Schmidt-Bleek et al. 2012b))

A more thorough analysis of the expression of inflammatory, angiogenic and osteogenic factors after 4 and 7 days of bone healing in the haematoma and callus tissue in the fracture gap shows that the pattern changes over time and that a mechanically induced delayed healing clearly influences these three aspects of healing (Fig. 4). (For a detailed description of these experiments please consult (Lienau et al. 2009; Lienau et al. 2010; Schmidt-Bleek et al. 2012a; Schmidt-Bleek et al. 2012b)). If the age of the haematoma is of importance for the cytokine pattern and this cytokine pattern is guiding the healing process, then a disturbance of the haematoma with the removal of specific factors will disturb bone formation in a healing situation.

## Can the haematoma turn into a hindrance for healing?

Apparently, the formation of fracture haematoma is crucial for the initiation and the success of the secondary fracture healing process. It would be expected that in closed diaphyseal fractures managed by closed reduction, followed by stabilization with external skeletal fixation or splint or cast coaptation, that the contribution of the fracture haematoma to fracture healing should be unperturbed. However, open reduction and internal fixation of closed fractures will inevitably disrupt the fracture haematoma, as would debridement and irrigation of open fractures. Although open fracture reduction in human trauma patients is a routine in clinical practice, the influence on fracture haematoma disruption or removal on subsequent fracture union has not been well studied. Moreover, since the surgical treatment of fractures is often delayed for days or weeks because of patient, local soft tissue or other factors, then the interval until surgery and haematoma disruption is another variable to be considered. Therefore, a pilot study in sheep was performed in which the fracture haematoma associated with a tibial osteotomy was removed after 4 or 7 days, respectively, to observe the hypothesized negative effect on the early stages of fracture healing (Lienau et al. 2009; Lienau et al. 2010).

A total of 19 female Merino mixed breed sheep (2.5 years old) with a mean weight of 67.3 kg (±5.5 kg) were randomly divided into three groups. All animal experiments were carried out according to the policies and principles established by the Animal Welfare Act, the NIH Guide for Care and Use of Laboratory Animals and the national animal welfare guidelines. The study was approved by the local legal representative (G 0224/01 and G 0172/04). In all 19 animals a standardized mid-shaft transverse osteotomy of the right tibia, was stabilized with a stable uni-lateral six pin external fixator, as previously described (Epari et al. 2006; Schell et al. 2005). The osteotomy mimics an open fracture with soft tissue injury and devascularisation at the fracture side. The stable external fixation leads to uneventful bone healing within nine weeks (Epari et al. 2006). During surgery the animals receive bolus injections (i.v.) of 0.25 mg/75 kg body weight Fentanyl (Jannsen-Cilag GmbH, Neuss, Germany) every 30 min. Prior to surgery the animals received a 75 μg Fentanyl patch (Durogesic® 75 μg/h, Jannsen-Cilag GmbH, Neuss, Germany) and were s.c. injected with Flunixin (Finandyne®, IntervetDeutschland GmbH, Unterschleißheim, Germany) according to their bodywheight for at least 3 days post surgery.

A second surgical procedure was performed for the removal of the fracture haematoma after 4 days (group D4, *n* = 6 sheep) or 7 days (group D7, *n* = 6 sheep) with wound-reclosure. Haematoma were removed and the fracture ends were refreshed during the procedure which was concluded by saline irrigation to ensure complete removal of the original haematoma. The time points of four and 7 days were chosen according to clinical practice when definitive osteosynthesis is usually performed. The remaining seven sheep did not undergo a second surgical procedure and formed the control group (Group C). The sheep were sacrificed two weeks postoperatively. Thus in all groups the healing progress was analysed histologically two weeks after osteotomy. After sacrifice, the tibiae were explanted for histological analysis and the callus regions were sectioned into 3 mm slices in the frontal plane. The slices were decalcified in EDTA, dehydrated with alcohol and xylol, embedded in paraffin and cut into 4 μm-thick histological sections. Histological analyses were performed on Movat Pentachrome stained sections, which allowed a distinct and colorful contrast between the different tissue types: fibrin is stained in different shades of red, cartilage is stained deep green, fibrous connective tissue is stained in light green-blue and bony tissue is stained in yellow. Furthermore, cells, e.g. osteoblasts, osteoclasts, inflammatory cells, can be easily differentiated.

**Fig. 5:**
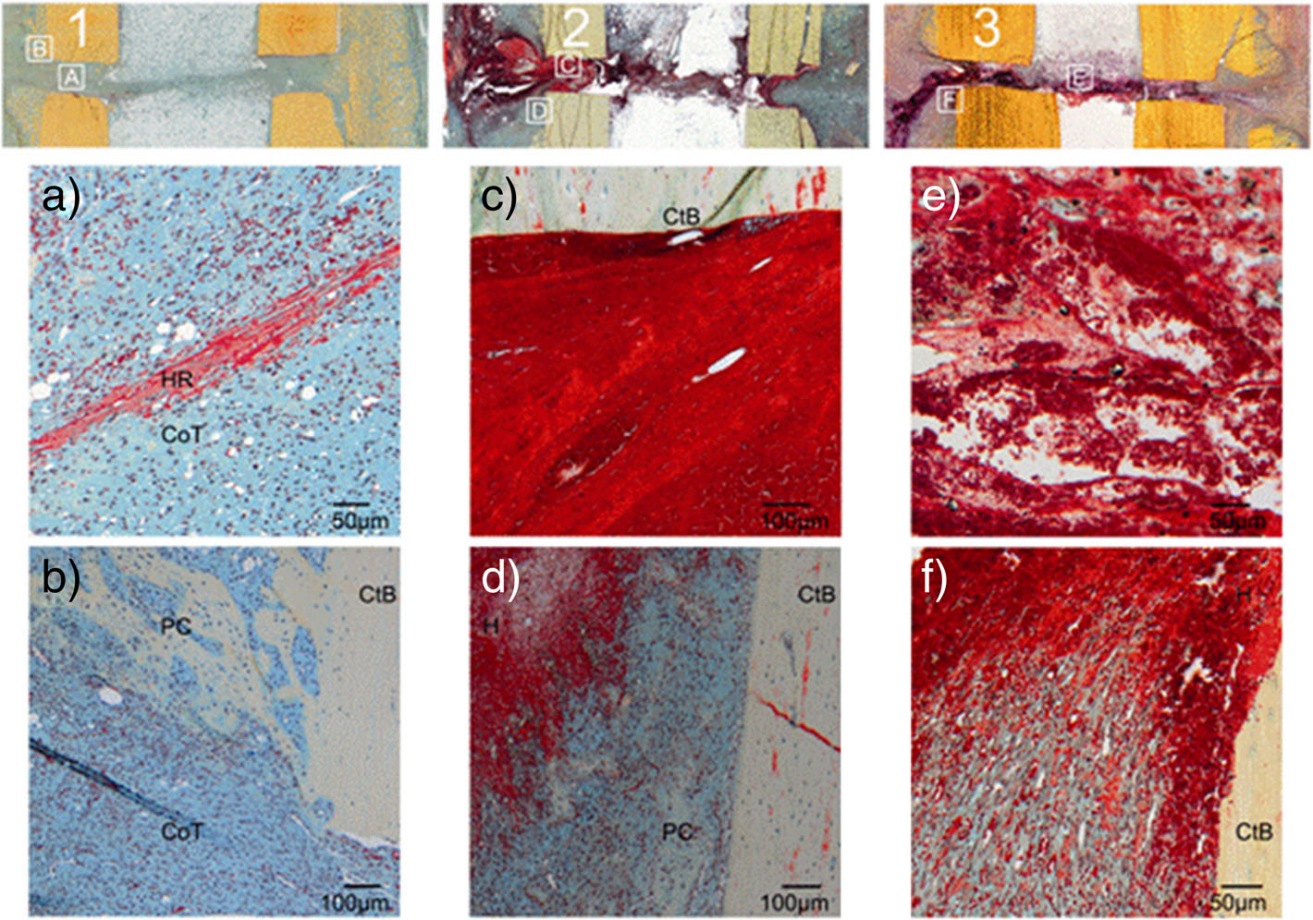
Movat Pentachrome staining 14 days post surgery of the control group and the haematoma removal groups (D4, D7). The control group shows the physiological healing pattern (1) while group D4 (2) and group D7 (3) are characterized by prominent haematoma remnants and a delayed periosteal callus formation (overview top). The squares in the overview indicate locations of magnifications shown below for each group. **a**, **c**, and **e** depict a comparison of the haematoma remnants in the osteotomy gap. Note the progressed organization of the haematoma remnants (HR) in the control group (**a**), where connective tissue (CoT) predominates in the osteotomy gap. At the two weeks time point this connective tissue represents the normal bone healing development and indicated the maturation of the haematoma towards the soft callus phase. In groups D4 and D7 the haematoma is still unorganized with dominating erythrocytes and only a small amount of fibrin fibers without noticeable orientation (**c**, **e**). **b**, **d**, and **f** show the progression of the periosteal callus (PC) with respect to the osteotomy gap. While the newly formed woven bone in the control group (**b**) developed along the cortical bone (CtB) up to the original gap, this is neither seen in group D4 (**d**) nor in group D7 (**f**)

This study showed that an undisturbed fracture healing can be characterized by a rapidly progressing periosteal woven bone formation with only remnants of a well organized fracture haematoma after two weeks of healing (Fig. 5). On the other hand, haematoma removal after 4 or 7 days induced a significantly higher fraction of haematoma.

**Fig. 6:**
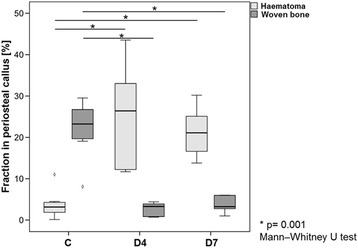
Haematoma and woven bone fraction in the periosteal callus area. Note the reverse ratio of haematoma and woven bone tissue in the control group and the treated groups with a significantly higher fraction of bone and a significantly lower fraction of haematoma in the control group

Histomorphometrical evaluation revealed a significantly lower fraction of newly formed woven bone, which was seldom found in the proximity of the fracture gap and never in the direct proximity of the haematoma (Fig. 6).

The removal of the haematoma after 4 or 7 days of healing sets a new inflammatory impulse (seen in the previous paragraph in the expresseion data and cellular composition). Since during physiological healing, after only 24 h the anti-inflammatory signalling increases, terminating the pro-inflammatory burst (Schmidt-Bleek et al. 2012b), this new inflammatory impulse could be equalized to a prolonged inflammation phase. A prolonged pro-inflammatory reaction has been shown to delay bone healing (Kovach et al. 2015; Lienau et al. 2010; Schmidt-Bleek et al. 2012a). This indicates that a delayed intervention in a clinical situation leading to a secondary haematoma in the fractured region may delay the healing process through the prolongation of the pro-inflammatory phase. It is conceivable that haematoma removal in elderly, multimorbid patients might eventually result in a delayed union, or even a non-union (Gibon et al. 2016; Sattler and Rosenthal 2016). Even the young healthy sheep that were included into the study showed a distinct delay in the early healing. Whether they could make up for the initial delay during a longer healing period remains speculative but seems probable.

## Clinical indications that the early inflammatory reaction is essential for a successful healing outcome

The conditions in an early fracture haematoma are not favourable for cells because the pH value is lowered due to the anaerobic energy turnover, the potassium and sodium concentrations are high, and blood supply is disrupted so that the environment is hypoxic (Street et al. 2000). These conditions are well tolerated by some immune cells. Among them the M1 macorphages which instantly switch to an anaerobic energy supply and remain active (Fangradt et al. 2012). Also T cells are able to stay functional (Gaber et al. 2009). The ability to thrive under these harsh conditions highlights the importance of immune cells during this healing phase. Investigation of cultured haematomas gained from the femur of patients undergoing a total hip arthroplasty allowed the monitoring of immune cells present during the early stage of bone healing. These studies confirmed the adaptation of immune cells via the expression of angiogenic factors, chemoattractants and pro-inflammatory molecules. In addition the restriction of the oxygen and nutrient supply selectively enhanced the survival of lymphocytes (Hoff et al. 2013). It is nowadays well established that in the early phase of healing cell mediated immune functions are essential for removing necrotic debris, for promoting angiogenesis, for recruiting cells to the site of injury, in short, to initiate repair/ regeneration. However next to the upregulation of the inflammatory reaction the timely termination of this reaction is also important, leading to a local increase of induced regulatory T cells that suppress adaptive immune responses within the fracture callus (Einhorn and Gerstenfeld 2015). A functional immune system therefore is important for bone healing. This led us to investigate bone healing in immunologically impaired patients. Indeed, cells within the fracture haematoma of immunologically restricted patients showed an unproportionally strong pro-inflammatory reaction, leading to an inadequate response to local hypoxia and resulted in a decreased osteogenic differentiation (Hoff et al. 2011). The recognition of the importance of the immune cells for the bone healing process lead to possible treatment options harnessing the immune reaction by using immune modulatory strategies to enhance bone healing (Mountziaris and Mikos 2008; Mountziaris et al. 2011). A recent overview is given by Loi et al. (Loi et al. 2016).

## The orchestrating role of the fracture haematoma

The early phase of fracture healing is dominated by the fracture haematoma. The complex microenvironment of the haematoma is of great importance for migrating cell populations. The maturation of the haematoma during the fracture healing process changes this microenvironment (Brighton 1984; Cruess and Dumont 1975). Starting an inflammatory response is an energy-intensive process. At this stage macrophages (M1) rapidly switch from a resting state to a highly activated state, using glycolysis and high glucose uptake to cover their energy demand and to produce pro-inflammatory cytokines (O’Neill and Hardie 2013). Due to the blood vessel disruption, the region becomes hypoxic forcing active cells into an anaerobic energy supply with subsequent accumulation of lactat (Komatsu and Hadjiargyrou 2004). In consequence, the early fracture haematoma not only shows challenging energetic conditions but also a low pH. The pH is changing from acidic through neutral to slightly alkaline during the initial phase of healing (Brighton 1984; Cruess and Dumont 1975). Granulocytes, which invade the fracture haematoma upon injury, have a life span of about 12 h. Upon apoptosis they release an oxidative burst, which could damage cells involved in healing. In summary, the conditions in the early haematoma are not ideal for progenitor cells (Street et al. 2000). The haematoma matures over the healing period and already after 24 h the anti-inflammatory signalling increases, to end the pro-inflammatory burst (Schmidt-Bleek et al. 2012b). With the progressing differentiation of the haematoma, the factors necessary for precursor cell differentiation probably come to the fore. By the removal of haematoma tissue, essential factors for periosteal and bone marrow cell proliferation and differentiation and finally bone healing are severely reduced or withdrawn. Disturbing the original haematoma may therefore delay cell differentiation and consecutively woven bone formation. Tachibana (Tachibana et al. 1991) demonstrated that the capability of fracture haematoma to induce ectopic woven bone formation depends on the age of the haematoma. A mature haematoma is supposed to contain more factors and cells essential for fracture healing than a fresh one. The secondary haematoma, formed after removal of the original fracture haematoma, would equal peripheral blood and is likely to provide a different microenvironment compared to the original (removed) fracture haematoma. Studies showed differences between the fracture haematoma and the peripheral blood, especially regarding the immune cell population (Schmidt-Bleek et al. 2009; Schmidt-Bleek et al. 2012a; Schmidt-Bleek et al. 2012b). Therefore, the initial fracture haematoma and its surrounding tissues evolve and develop in tight interplay. After removal of the fracture haematoma, the finely adjusted composition of haematoma and adjacent tissue is severely disturbed. The matured initial haematoma is replaced by a new and therefore naïve haematoma, whose composition does not fit to the healing stage of the surrounding tissues and which in the beginning is quite detrimental to progenitor cells. Additionally to the removal of essential cells and factors by harvesting, this evolutionary mismatch between new haematoma and surrounding tissue may further delay the fracture healing process.

## Conclusion

In todays clinical routine the healing potential of the initial fracture haematoma is still not fully recognized. In recent years the awareness of the regenerative potential of the early fracture haematoma rose which is documented in the relatively new MIPO strategy that shows good results in fracture treatment – a strategy that does not disturb the haematoma more than necessary and is never removing parts of it.

Our knowledge about the rising importance of osteoimmunological aspects of the healing process also supports the important role of the intial haematoma as the first influx of inflammatory cells and the ensuing cytokine pattern initiating cell recruitment towards the injured tissue are a direct result. Future treatment recommendations should include the reference to the important regenerative function of the initial fracture haematoma and thus the recommendation to keep the initial haematoma whenever possible during internal fixation of fractured bones.
